# The Help Question

**Published:** 1870-04

**Authors:** Ben. H. Pratt


### THE HELP QUESTION.

### Another Plea for Bridget.

BY BEN. H. PRATT, A. M.

By whose first disobedience has this fruit of Domestic Help, once so fair to look upon, but now so bitter in our maw, been thrust upon us? However great may have been the skalawagism of our forefathers—whatever of ills they may have bequeathed us, (although I am told it is highly improper to defame our so-called sainted progenitors,) this Help Question is not one of their legacies. They lived, loved, delved and died, wholly innocent and unconscious of the misery in store for their descendants, arising from the machinations of ubiquitous Biddy. Their social code was such that this modern improvement was not a necessity—ours, unfortunately, is not so ordained. We alone are the authors of this our latter day misfortune. And how in the world did we ever come to make such a fatal mistake? This is how.

Down the road a little way, just below the blacksmith's shop, on a farm of four or five hundred acres, lived, with her estimable husband and two buxom beauties of daughters, Mrs. John Smith. Blessed with good health and freedom from all nonsensical ideas of style and grandeur, she affected no display in matters domestic beyond the annual dinner or tea company for her neighbors of Rillville, at which she with modest pride presided, and in honor of which her choice old wedding china and snowy table linen got an airing. She lived content—loved her good man—taught her dear girls in the good old school and house-lore of primitive common sense—did her own work, her daughters lending helping hands—blessed her stars for what she had, nor once repined for what she had not.

On speculation bent, with a prophetic eye upon the profits of a prospective sawmill which, when driven by the creek that no Rillvillian had ever turned to other purpose than a watering place for cattle, should rip a rapid fortune out of plenteous pines, came, from Boston town, one Abel Jones, with notions far above the simple folk of Rillville. The purchase of sufficient grounds for milling purposes, a tract of timber land and a site for a residence, soon followed; and thereupon arose a mill, the pride of Rillville, and a dwelllng, the envy of all the neighborhood. Then came from Boston such loads of household stuff as set the villagers all by the ears; carpets of brussels! chairs and tete-a-tetes with cushioned springy seats! tables with marble tops! and crowning all, a piano!! Not many days elapsed, when, to the door of Abel Jones, Esq , drove up the evening coach from Boston, and therefrom alighted Mrs. Abel Jones, her daughter Ruth, now turned sixteen, Abel, Jr., just into trowsers, and a stoutish girl with features plainly Celtic, and evidently a servant.

However high above the villagers the Joneses seemed at first, not many months slipped by before a degree of sociability sprang up between the new-comers and the better Rillville families, and access to the great house was had, and that knowledge of its domestic arrangements acquired for which the dwellers in little towns are proverbially apt.

It was then and there that over Mrs. Smith there seemed to come, all at once, a sad unveiling of the past and all its household cares. How great a slavery she'd endured, till now had never crossed her mind. To see that Mrs. Jones, the live-long day, sit at her window with her work or book; or with her daughter Ruth, slip out for a walk regardless of the dinner-work, was gall to her and the fair damsels Smith. Then she compared the fair hands of the Joneses with the rough and red that marked her daughters' and her own. Twouldn't never do. She'd see Smith about it this very night. A gal to do the work like Mrs. Jones' she *would* have, and Sue and Beckey shan't hide their hands afore no sich folks as Mrs. and Ruth Jones. The pianner she didn't keer about jest now, but by an by she'd show that lilly fingered set that *her* gals could thump a tune as good as Boston folks.

Of course a Domestic was duly installed in the house of Smith. The sequel's plain. Mrs. John Smith has her representatives the states over. Joneses, with strange notions of style, have come to every hamlet and inoculated all the Smiths with their ideas of living. We don't know whom to censure the more, the Joneses or the Smiths, nor does it matter. We're all Smiths at last, and all we've got to do now is, to be the best Smiths we can be under the peculiarly afflicting circumstances. We have ceased to be independent—have surrendered our right to do our own household work in our own way—have established Bridget as a necessity—and all that remains for us to do is, to see what ails the relationship between mistress and maid, and endeavor, in a measure to ameliorate matters.

In a previous article for this magazine, I endeavored to set forth the grievances of Madam and the escape therefrom, as seen from her standpoint, and said things too true to be popular. In this article—BISTOURY willing—I propose glancing at Mistress through Biddy's goggles—of viewing, briefly, the Help Question from the standpoint of the Domestic.

With few exceptions, the days of the years of the life of a housekeeper are full of trouble, and to no one source can so much of dire discomfort be ascribed—so much of vexatious unrest be attributed—so much of excusable and inexcusable impatience be imputed—as to this one ever rising, never allayed, now quiescent, now turbulent, now rabid spectre, "our kitchen girl."

That many matronly tempers have been spoiled—many wifely dispositions soured—many feminine graces masculinized—many comely faces prematurely furrowed—all by this grim and ghastly gnome below stairs—nobody for a moment doubts. So at least we are daily told, so we almost daily see—for this we fain would heave the sympathetic sigh; but, unfortunately, sighs don't help matters—never did help any matter. Reason's a heap the better plaster for this our home sore; let's apply it tenderly.

Your hired girl may be ignorant, but no fool ; she may not belong to any "Cooks' Protective Union," but enjoys the privilege of discussing you, pro and con, in her own coterie of choice spirits, nevertheless; you may deem her loyal to your government—bending at your nod—shrinking at your censure; but she shakes her fist, mind you, behind your back, and there's a little attic room in somebody's house where all your faults, few or many, are compared—and fairly, too—with all your excellencies, and a balance struck. You are known for just what you are by every Domestic in your neighborhood. No friend, however intimate, knows you half so well as she whom you deem scullion. Are you a kind and considerate Mistress; you are nowhere more cheerfully and soulfully honored than in that searching tribunal. Are you mean and exacting and over-bearing; make up your mind that you are but sowing seeds of trouble that will some day spring up rank and strong, and choke every blade of domestic content that is struggling for existence in the soil of your home circle.

Do kindly disposed, charitably inclined, reasonable mistresses long wait servantless? Can your she tiger get a decent servant to remain with her at any cost? Have you never pondered upon this fact? Does not this alone prove how well you are known—how justly you have been rated—how righteous a sentence has been passed upon you? Flatter not yourselves, my worthy Madams Smith, that your Domestic is too dull to penetrate the mask you don't always remember to adjust carefully before you confront her. An intercourse as constant and intimate as yours and hers makes her the most dangerous spy that society affords. You are studied, watched, tested, tried, in countless ways, at unsuspected times and under the most unfortunate circumstances, perhaps, for you. The ordeal a mistress must go through under one year's espionage by even the mildest of servants, is a searching one. Few can emerge from it unscathed. Is it strange, then, that many mistresses are constantly changing maids, when hate or pity or contempt or fear are oftener begotten than love or sympathy or esteem or admiration or honor or even respect between the two? When your servant learns that you regard her simply as a slave, or a machine, and the amount and quality of the work turned off the only end you seek, you are a harsh mistress, though not a word or act of yours has been unkind. She sees you indifferent to the excellence of some difficult feat of cooking or other duty, over which she has taken special pains, and your ingratitude touches her sorely. She asks a holiday, or claims an evening, or an hour or two after work in the afternoon, and your frown shows your unwilling consent; will she work as cheerfully and carefully the next day? Is it human nature to do well for those that treat us ill? Is it not natural to make almost any sacrifice for those who interest themselves in our welfare? Are the relations between mistress and maid so different from those between master and man that the treatment must differ so widely? Does not the maid-of-all-work know that she is the most unremitting worker in society, for the least remuneration? What class of workers rise at five and toil till seven and often ten at night, Sundays not excepted, with no nooning, for from five to ten dollars per month and board? She thinks of this when she asks an occasional holiday, or a favor of any kind. Does Madam think?

We may imagine ourselves excusable in supposing that one who will do so menial a work as does the genus "help," must, of necessity, possess a mind below that of the average woman; but it is by no means so. Necessity sometimes compels us into stations we despise. The class from which our domestics come is poor, unable to afford their children an education fitting them for any higher sphere. Thrown into service when young, by stern necessity, they are fitted for no other position, and find our kitchens the only avenues open to them for obtaining a livelihood. Upon this ignorance we are far too apt to impose, imagining that sense and sensibility can never go hand in hand with ignorance. This however is an error, for a fault is as quickly seen—a wrong as sorely felt—an injustice as keenly recognized by those of low degree as by our own more felicitous kind.

Their low estate, too, seems to us at first sight as incompatible with independence or resentment of wrong, whereas they are, in this respect, our superiors, knowing that we must employ them; and reasoning far better than we upon the scarcity of help. They appreciate the fact that none will enter their domain except upon compulsion, the higher avenues to a livelihood being taken by all whom a little better education has fitted for more agreeable avocations and better remuneration. They reason well upon the fact that the supply of domestic labor can never exceed the growing demand, and hence a good girl can afford to leave place and mistress upon proper provocation, without anxiety as to speedily obtaining another. This is a point which seems so myterious among mistresses.

In a general way, I do not know of any test one can so well and profitably apply in determining her duty to her servants, as that of imagining herself in their position, and then comparing the treatment she is likely to get with that which she desires. It is in observations made from the kitchen rather than the parlor that we can make a just estimate between these two powers. Women, in command, are apt to be inconsiderate—to forget that there are duties due toward, as well as from, the employed, aside from the paying the market value of the latter's services. A few grains of charity thrown into the details of mistresses' miseries, would affect them much for the better.

Upon my word, I fear that an unbiased jury after weighing the arguments of a coterie of mistresses and domestics, would render a verdict not quite so complimentary to the former as they would like; and I repeat, that this Help Question can never approach anything like a settlement, until the average mistress and average maid have well weighed their respective relationships, and stricken several degrees of altitude from the false notions of both.

In closing, the writer would have it clearly understood that he is offering no plea for the countless applicants for work that crowd our larger cities, and infest our "intelligence offices;" with no ability and if possible less character. With such, patience and forbearance is thrown away. We can only pity the harborers of such *canaille.* Our plea is for the average "hired girl," of whatever nationality, and who is respectable in that she prefers to do a menial work rather than be dependent upon others, or accept the luring bait too often held out to such, the price of which is departed virtue and a lost womanhood.

---

A French chemist has discovered that a dress, made of green tarlatan, contained by weight, no less than 391.4 grammes of arsenite of copper (the green coloring substance), equivalent to more than half a pound of arsenic.

---

A man recently arrived in a town in Minnesota, bought a lot, built a house, and set up housekeeping within the space of forty-eight hours, and had a son and heir born to him before the first meal was eaten in the house.

